# Targeting Neph1 and ZO-1 protein-protein interaction in podocytes prevents podocyte injury and preserves glomerular filtration function

**DOI:** 10.1038/s41598-017-12134-8

**Published:** 2017-09-21

**Authors:** Amin Sagar, Ehtesham Arif, Ashish Kumar Solanki, Pankaj Srivastava, Michael G. Janech, Seok-Hyung Kim, Joshua H. Lipschutz, Sang-Ho Kwon, Ashish, Deepak Nihalani

**Affiliations:** 1 CSIR-Institute of Microbial Technology, Chandigarh, India; 2 Division of Nephrology, Medical University of South Carolina, Charleston, SC USA

## Abstract

Targeting protein-protein interaction (PPI) is rapidly becoming an attractive alternative for drug development. While drug development commonly involves inhibiting a PPI, in this study, we show that stabilizing PPI may also be therapeutically beneficial. Junctional proteins Neph1 and ZO-1 and their interaction is an important determinant of the structural integrity of slit diaphragm, which is a critical component of kidney’s filtration system. Since injury induces loss of this interaction, we hypothesized that strengthening this interaction may protect kidney’s filtration barrier and preserve kidney function. In this study, Neph1-ZO-1 structural complex was screened for the presence of small druggable pockets formed from contributions from both proteins. One such pocket was identified and screened using a small molecule library. Isodesmosine (ISD) a rare naturally occurring amino acid and a biomarker for pulmonary arterial hypertension was selected as the best candidate and to establish the proof of concept, its ability to enhance Neph1-CD and ZO-1 binding was tested. Results from biochemical binding analysis showed that ISD enhanced Neph1 and ZO-1 interaction under *in vitro* and *in vivo* conditions. Importantly, ISD treated podocytes were resistant to injury-induced loss of transepithelial permeability. Finally, mouse and zebrafish studies show that ISD protects from injury-induced renal damage.

## Introduction

Protein complexes actively participate in many biological processes and disease pathologies making them an attractive target for drug developers^1,2^. It was long believed that PPI interfaces are too large and not suitable for the binding of small molecules, which was one of the major reasons for slow progress in this field^2,3^. However, recent studies have shown that functional region in a PPI is small enough to be regulated by small molecules^1,3,4^. Almost all drug development using this approach has targeted “inhibiting” PPIs, where many of these drugs have entered various phases of clinical trials^2,5,6^. Mechanistically, majority of these drugs bind one target protein and inhibit its ability to form a functional complex with its binding partner, thereby modulating its downstream signaling events^2,4^. Many small molecules have been developed that inhibit various PPIs including the Ras-SOS1 complex that produces anticancer effects by targeting Ras oncogene^4,7^; small molecules LEDGINs were shown to inhibit LEDGF/p75-integrase binding^8^ and inhibit HIV replication; Verteporfin was shown to inhibit YAO-TEAD complex with anticancer properties^8,9^. It is to be noted that this approach has been used to develop several other potential PPI inhibitor drugs that are currently under clinical trials targeting a variety of cancers^5,6^; however, not much progress has been made in targeting other diseases. In this study, we show that rather than inhibiting a PPI, a molecule that will strengthen a PPI may produce similar therapeutic advantages in glomerular biology. We demonstrate this using two podocyte proteins Neph1 and ZO-1, whose structural complex was recently described by our group^10,11^.

Many renal diseases lead to the disruption of glomerular filtration barrier resulting in major loss of renal function and leakage of protein into urine, a condition that is commonly known as proteinuria^12–14^. The filtration barrier of a kidney is composed of three major cellular layers, which include, the fenestrated endothelium, glomerular basement membrane, and podocytes^14,15^. In the past decade, podocytes have gained significant attention since the structural integrity of their junctions also known as “slit diaphragm” is critical for maintaining glomerular filtration function. Podocytes are the primary target of many glomerular diseases and injury to podocytes progresses to ESRD (end state renal disease)^13,16^. Over the years many proteins including Nephrin, Neph1 and ZO-1 have been characterized that constitute the framework of podocyte junctions^11,15,17,18^. Interestingly, majority of these are junctional proteins and are critical for slit diaphragm integrity^14,19^. Although the exact functions of these proteins in podocytes is unclear, several studies now show that these proteins participate in many signaling pathways and mediate numerous interactions that are critical for podocyte function^12,19,20^. Importantly, genetic deletion of these proteins leads to podocyte effacement resulting in proteinuria and renal failure^18,21,22^. Although many interactors of Nephrin and Neph1 have been defined^17,20,22^, but how these interactions participate in maintaining podocyte function is not clear. Interestingly, injury to glomerulus has been shown to dissociate Neph1-Nephrin and Neph1-ZO-1 complexes and induce redistribution of these proteins from podocyte cell membrane to cytoplasm^10,23,24^. Thus these proteins and their complexes may define the structural integrity of slit diaphragm, which is prone to damage during glomerular injury.

Neph1 is a transmembrane protein whose primary structure consists of an extracellular domain containing five IgG like domains, a transmembrane domain, which is followed by a short intracellular domain^22,24,25^. Despite being widely distributed in tissues such as heart, liver, brain and kidney, the loss of Neph1 primarily produced a renal phenotype^10,22,24^. While the extracellular domains of Neph1 mainly contribute towards the structural framework of slit diaphragm^25–27^, the cytoplasmic domain of Neph1 has been shown to induce a signaling cascade that regulates actin cytoskeletal reorganization^10,13,28^. In addition, the cytoplasmic domain interacts with a number of proteins including, podocin, ZO-1, IQGAP1, CD2AP, β-arrestin, Nck, CASK, Grb2, α-actinin 4, synaptopodin, and Myo1c^15,17,18,25,28,29^. Although the exact functional significance of these interactions remains unknown, it is believed that these complexes may participate in maintaining podocyte actin cytoskeleton^13,28^. Studies have also shown that the PDZ binding domain of Neph1 that interacts with ZO-1 and Par 3 proteins, plays a critical role in Neph1 function^10,30^. Our previous studies showed that Neph1 interacts with ZO-1 in a dynamic fashion and this interaction is highly responsive to glomerular injury^10^. We also published the structural details of this interaction which provided novel insights into the nature of this complex. Based on these facts we hypothesized that the Neph1 interactions at podocyte cell membrane are critical for maintaining the structural integrity of podocytes and thus regulate podocyte function^11^.

This study was inspired by the fact that podocytes are primary target in many glomerular injuries that not only induce significant changes to their actin cytoskeleton leading to a process called “foot process effacement” but also the loss of slit diaphragm^13,14^. It has also been shown that loss of slit diaphragm is central to the process of glomerular injury^14,15^. Our previous report showed that the interaction between Neph1 and ZO-1 was lost in response to ischemic injury leading to podocyte effacement and proteinuria^10^. This prompted us to test a concept, where stabilizing this interaction will prevent podocyte injury and preserve podocyte function in response to glomerular injury. Since we previously published the structural details of Neph1 and ZO-1 interaction, we wanted to utilize this information to identify small druggable pockets in this interaction and screen molecules with ability to simultaneously interact with both the proteins. Indeed, the structural analysis of this interaction revealed several druggable pockets which were formed by epitopes contributed by both the proteins and were individually scored. Further, based on the best score, a pocket was selected and was used in virtual screening protocol to search for small molecules. This led to the identification of several candidate molecules, among which isodesmosine (ISD), a naturally occurring compound was selected and tested for its ability to enhance Neph1 and ZO-1 interaction. The results presented in this study show the applicability of this novel approach in identifying potential molecules such as ISD that may have the ability to enhance Neph1 and ZO-1 interaction (or interactions of other podocyte proteins), which may contribute towards protection of podocytes from injury.

## Results

### Identification of drug pockets and in-silico screening in Neph1 and ZO-1 complex

**Figure 1:**
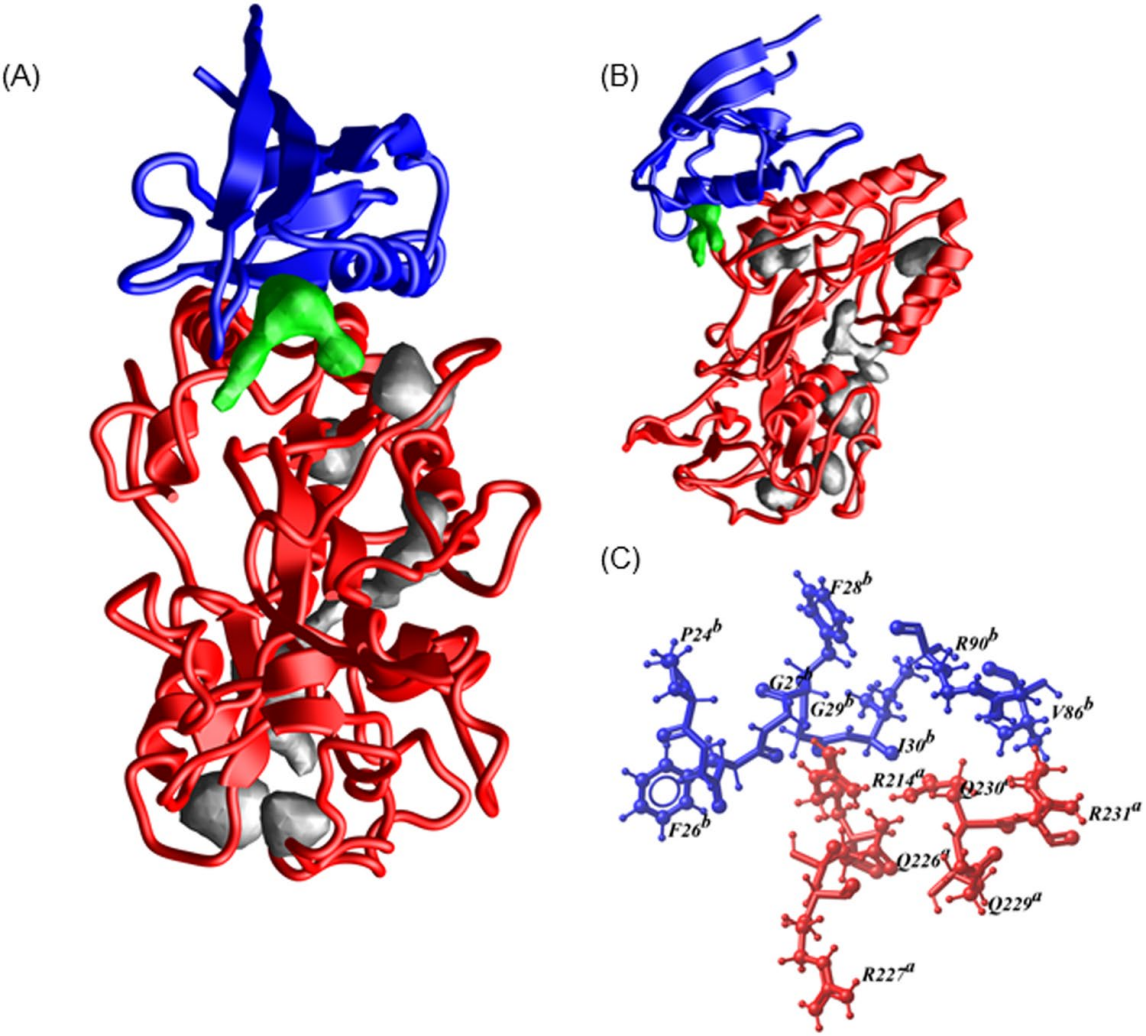
A druggable pocket is formed at the interface of Neph1-CD and ZO-1-PDZ1 complex. (**A**,**B**) Two orthogonal views of the pockets identified in the complex of Neph1 CD (red ribbons) and ZO-1-PDZ1 (blue ribbons). The pocket formed at the interface of Neph1 and ZO1-PDZ1 complex is shown in green, while all the other pockets are displayed in grey. (**C**) The *ball and stick* model shows residues from the interface pocket of Neph1-CD and ZO-1-PDZ1 complex that was used as receptor for docking simulations. The residues are labelled with a single amino acid letter code, followed by their position and the chain names *a* or *b* representing Neph1-CD and ZO-1-PDZ1, respectively. The image acquisition softwares are listed in the methods section. Final images were imported to power point, labeled and saved a tiff files.

**Figure 2:**
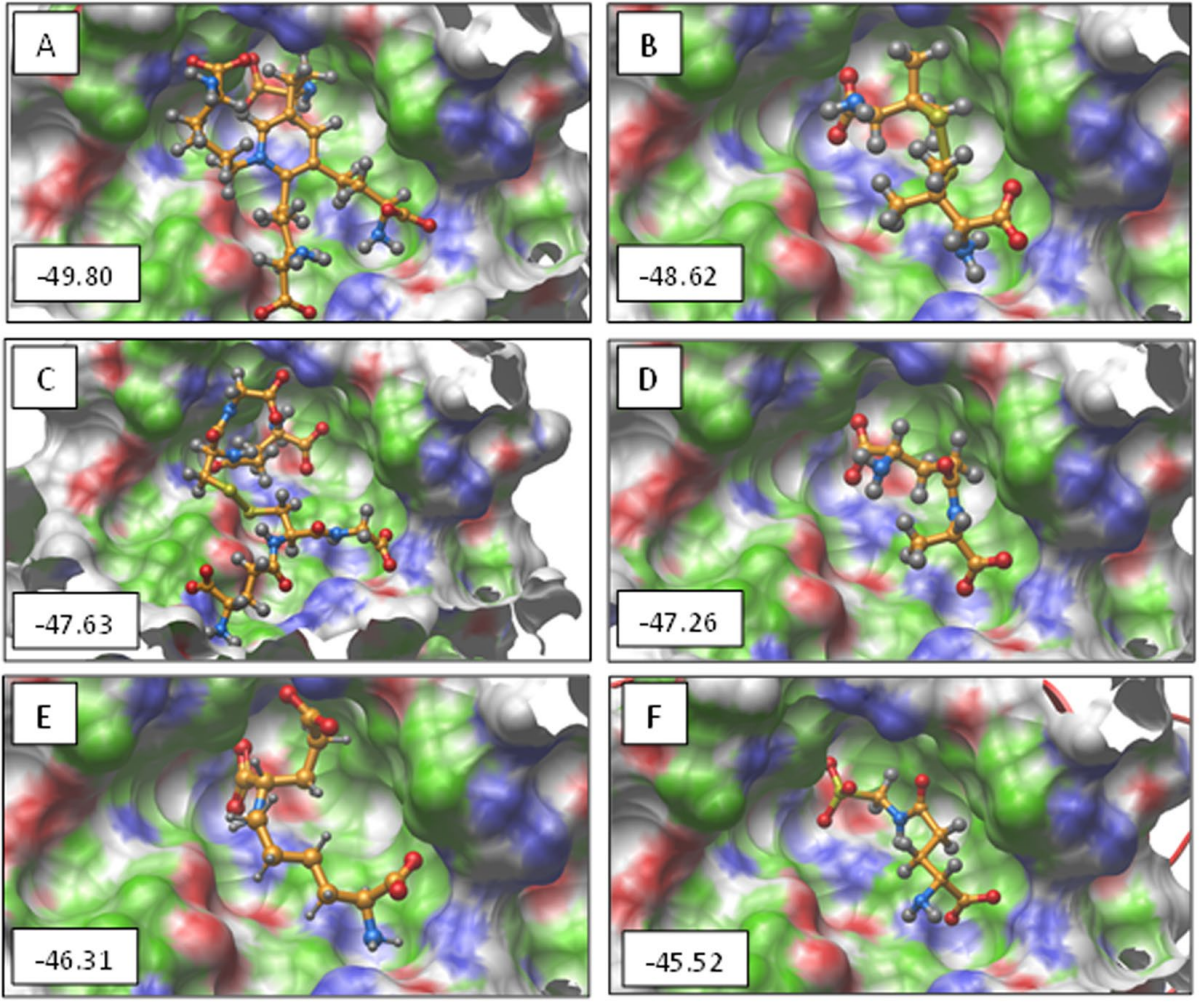
The virtual ligands screening using Neph1-CD-ZO-1-PDZ1 complex interface identified six molecules. The lowest energy docked poses of Isodesmosine (ISD) (**A**), d-penicillamine (**B**), l-glutathione (**C**), γ-glutamyl-l-alanine (**D**), l-saccharopine (**E**) and γ-d-GAMS acid (**F**) are shown in a *ball-and-stick* representation over the receptor pocket. To indicate the ligands binding property of receptor, the receptor surface is colored with green, red and blue representing hydrophobic, hydrogen bond accepting and hydrogen bond donating residues, respectively. The docking scores are displayed at the bottom left corner of each model. The image processing details are listed in the methods section and the final images were imported in power point and saved as tiff files.

**Figure 3:**
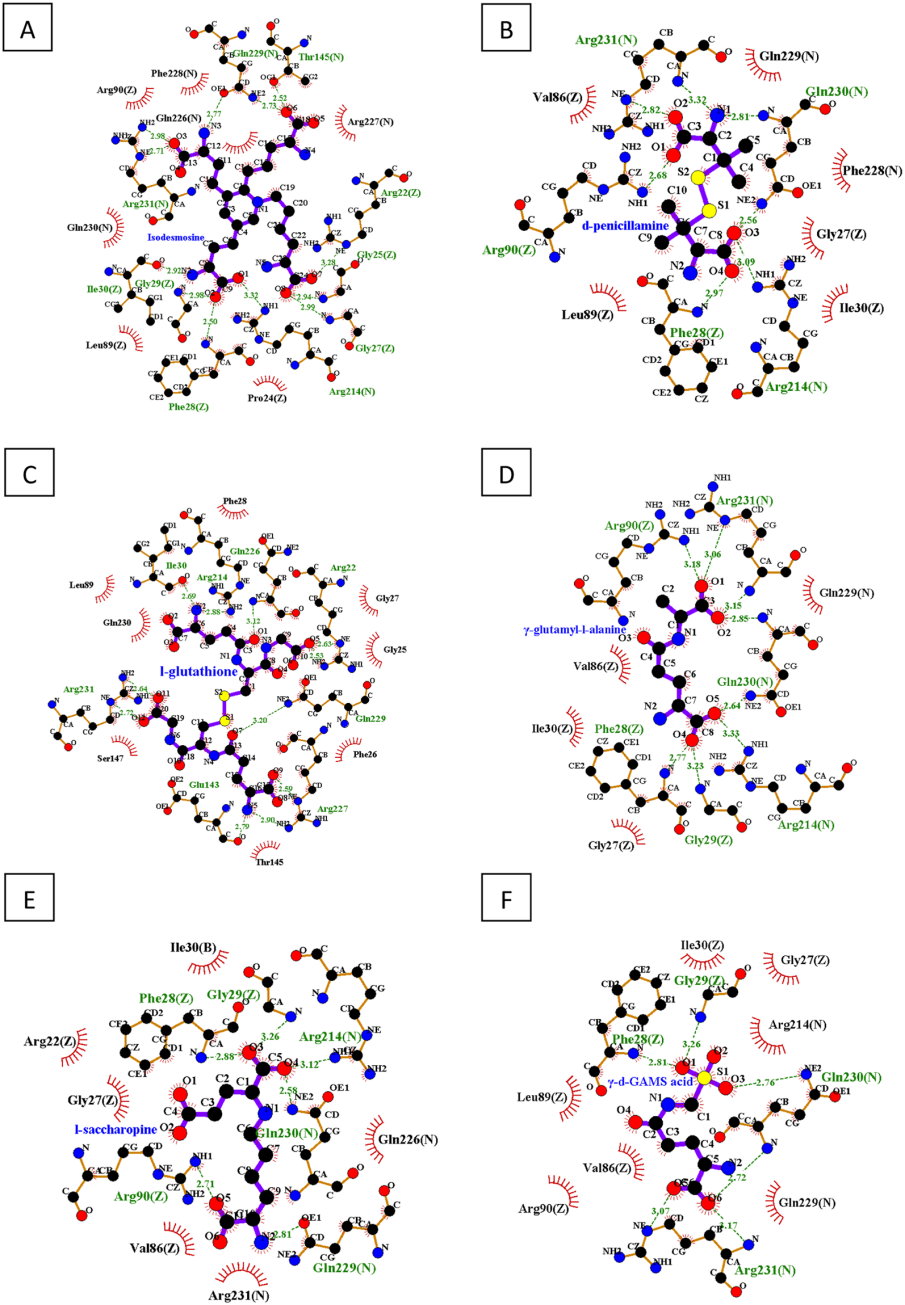
Interaction analysis of the best ranked compounds based on virtual ligand screening. Two dimensional representations of the interactions made by Isodesmosine (ISD) (**A**), d-penicillamine (**B**), l-glutathione (**C**), γ-glutamyl-l-alanine (**D**), l-saccharopine (**E**) and γ-d-GAMS acid (**F**) with receptor residues are shown. The *ball-and-stick* model shows residues involved in hydrogen bonding (green lines) with the ligand and the interatomic distances are shown in Å. The red spiked arcs represent residues contributing to hydrophobic interactions in the drug-receptor complex. Image processing details are listed in the methods section. The final images were imported to power point, labeled and saved as tiff files.

To test the hypothesis that binding of Neph1 with ZO-1 creates pockets where small molecules can be fitted, we used the ICM-PocketFinder program that predicted six pockets in the Neph1-CD-ZO-1-PDZ1 complex structure (Fig. 1A). Among these, only one pocket was formed where residues from both Neph1-CD and ZO-1-PDZ1 proteins contributed (Fig. 1A). The area and volume of this pocket were calculated to be 199.4 Å^2^ and 143 Å^3^ respectively with a non-sphericity value of 1.5. Overall, this pocket lied within the parameters of traditional drug targeted pockets. The residues contributed by each binding partner to the common pocket are: R(214), GQR (225–227), QQR (229–231) of Neph1 and GFGFGI (25–30), V(86), LR (89, 90) of ZO-1. A library of 800 amino acids and amino-acid derivatives was docked to this pocket using ICM 3.5. The original aim was to identify amino acids which bind strongly to this site and use those to synthesize peptides. However, we found that some of the individual amino acids had a very good docking score and occupied the pocket completely. The six molecules with the highest to lowest docking score were ISD, d-penicillamine, l-glutathione, γ-glutamyl-l-alanine, l-saccharopine and γ-d-Glutamyl amino methyl sulphonic (GAMS) acid with scores of −49.90, −48.62, −47.63, −47.26, −46.31 and −45.52, respectively. We analyzed the interactions made by these molecules with the receptor site using LigPlot^+^. The lowest energy poses and 2D representations of the interactions made by these compounds are presented in Figs 2 and 3, respectively. Analysis of the interactions made by ISD, the best ranked compound in the lowest energy conformation, revealed that ZO-1 residues Arg-22, Gly-25, Gly-27, Phe-28, Gly-29, and Ile-30 and Neph1 residues Thr-145, Arg-214, Gln-229 and Arg-231 made hydrogen bonds with ISD. Additionally, ISD formed salt bridges with Arg-22 and Arg-90 of ZO1 and Arg-214 and Arg-231 of Neph1, and made hydrophobic contacts with residues Leu-89 and Pro-24 of ZO-1 and Gln-226, Arg-227, Phe-228 and Gln-230 of Neph1. These interactions are tabulated in supplementary Table 1 and depicted in Fig. 3. This encouraged us to test the ability of ISD and other compounds in stabilizing the complex of Neph1-CD and ZO-1-PDZ1 using biophysical and biochemical assays.

### ISD stabilizes the Neph1-CD and ZO-1-PDZ1 complex: a gel filtration analysis

**Figure 4:**
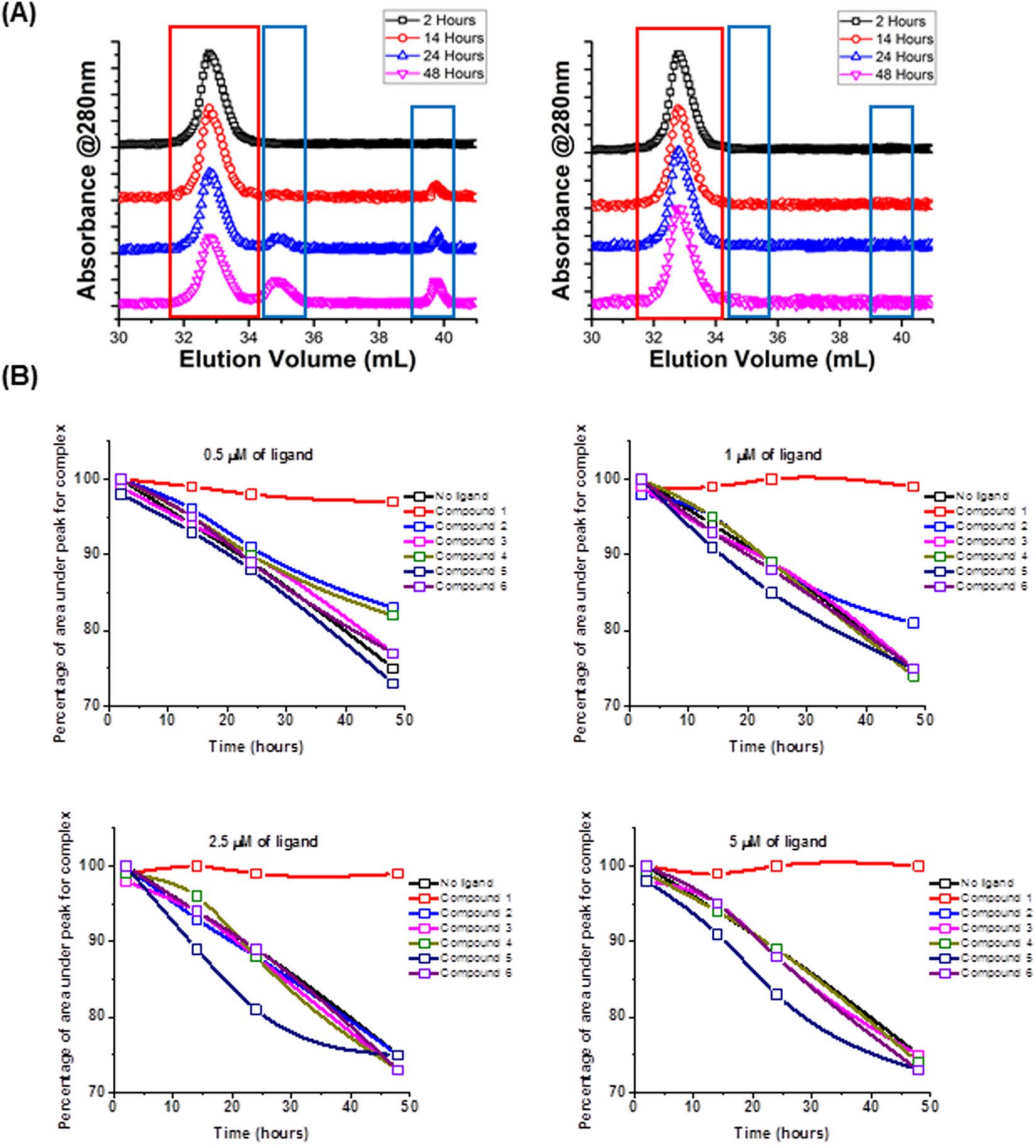
ISD stabilizes the Neph1-CD and ZO-1-PDZ1 complex. FPLC profiles of a binary mixture of purified Neph1-CD and ZO-1-PDZ1 proteins. (**A**) The plotted elution profiles show that Neph1-CD forms an efficient complex with ZO-1-PDZ1, which dissociates into individual proteins with further incubation (smaller peaks following the larger peak of 1:1 complex indicated with red boxes) in the absence of ISD (left panel). In contrast, the presence of ISD does not allow dissociation of Neph1-CD and ZO-1-PDZ1 complex (right panel). (**B**) Change in the area under the peak corresponding to the binary complex of Neph1 and ZO-1 in the absence or presence of various concentrations of ligands (Compound 1: ISD; Compound 2: d-penicillamine; Compound 3: l-glutathione; Compound 4: γ-glutamyl-l-alanine; Compound 5: l-saccharopine; Compound 6: γ-d-GAMS acid) were plotted and showed that the area under peak significantly decreased over time with all the compounds tested except ISD. Data obtained in the excel format was imported to Origin Lab software and the elution profiles were plotted. The final images were transferred to power point, labeled and saved as tiff files.

Earlier, using a Size Exclusion Chromatography (SEC) assay we reported that the 1:1 complex of Neph1-CD and ZO-1-PDZ1 dissociates slowly at 4 °C^11^. To test the ability of screened compounds including our best scoring compound ISD in promoting and stabilizing the binding between Neph1-CD and ZO-1-PDZ1 we used a similar SEC assay. In the first experiment, Neph1-CD and ZO-1-PDZ1 proteins were mixed in a 1:1 molar ratio (5μM) and incubated in the absence or presence of ISD. Size exclusion chromatography was performed at each indicated time intervals (2, 14, 24 and 48 h). Gel filtration analysis in the absence of ISD suggested that Neph1-CD-ZO-1-PDZ1 migrated as a higher molecular weight complex with a smaller elution volume of 33 ml and the individual proteins Neph1-CD and ZO-1-PDZ1 migrated at higher elution volumes of 35 and 40 ml (Fig. 4A). Expectedly, time-induced dissociation of Neph1-CD-ZO-1-PDZ1 complex was apparent after 14 h and persisted at 48 h (Fig. 4A). In contrast, no dissociation of Neph1-CD-ZO-1-PDZ1 complex was visible when ISD was added to this mixture (Fig. 4B). This provided first evidence that ISD has the ability to stabilize the Neph1-CD and ZO-1-PDZ1 complex.

A similar experiment as above was performed where 5μM of Neph1-CD and ZO-1-PDZ1 proteins were mixed with different concentrations (0.5, 1, 2.5 and 5 μM) of either isodesmosine (ISD), d-penicillamine, l-glutathione, γ-glutamyl-l-alanine, l-saccharopine or γ-d-GAMS acid. The analysis of SEC results is presented as percentage of area under the peak representing the binary complex as a function of time (Fig. 4B). Five of the six compounds tested showed a significant gradual decrease in area under the peak over time, while area under the peak did not change over time in the presence of ISD (Fig. 4B). This indicates that there was minimal time-induced dissociation of Neph1-CD and ZO-1-PDZ1 complex in the presence of ISD, providing further evidence for the role of ISD in stabilizing this interaction.

### ISD enhances the interaction between Neph1 and ZO1

**Figure 5:**
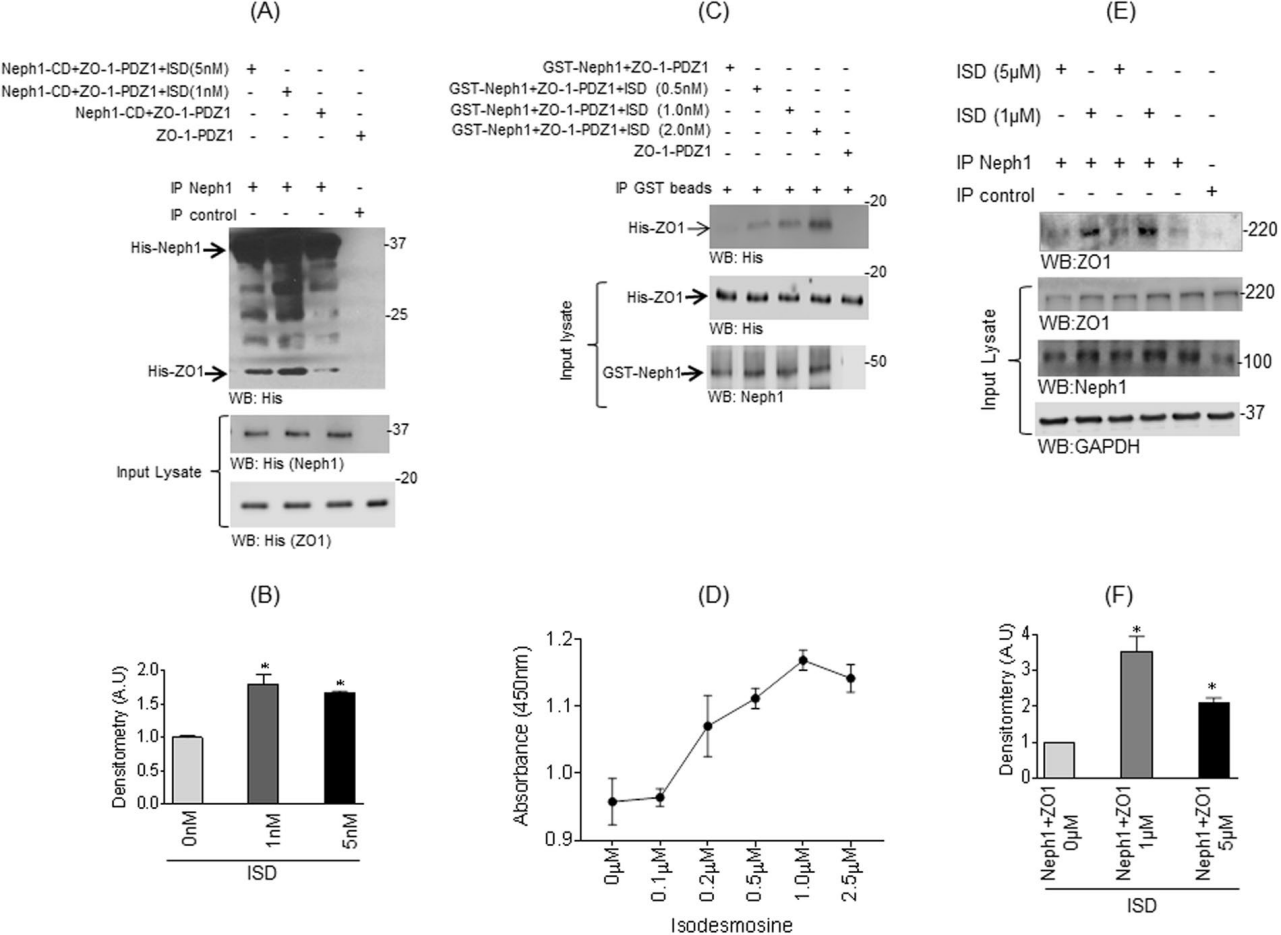
Binding between Neph1 and ZO-1 is enhanced in the presence of ISD. (**A**) Neph1-CD and ZO-1-PDZ1 purified proteins were mixed at equimolar concentrations in the presence or absence of ISD and Neph1 immunoprecipitation shows increased binding of ZO-1 as detected by western blotting using His antibody. (**B**) Densitometric analysis shows increased Neph1-CD and ZO-1-PDZ1 binding in the presence of ISD (1 nM and 5 nM). (**C**) Similar results were obtained when recombinant GST-Neph1 and His-ZO-1-PDZ1 proteins were incubated in the absence or presence of ISD, where Neph1 was pulled down using GST beads and bound ZO1 was detected using His antibody. (**D**) ELISA was performed to evaluate ISD mediated increase in Neph1 and ZO1 binding. An increase in the Neph1-CD and ZO-1-PDZ1 binding was observed with increasing amounts of ISD. (**E and F**) ISD enhanced the interaction between endogenous Neph1 and ZO-1 in cultured podocytes. Following treatment with ISD, the cells were lysed and immunoprecipitated with Neph1. Analysis of the complex by western blotting showed increased ZO-1 binding in the presence of 1 µM ISD.

Since virtual screening suggested that ISD binds at the interface of Neph1-CD and ZO-1-PDZ1 interacting site, and stabilized this interaction (Fig. 4A,B), we wanted to further investigate whether the addition of ISD enhances this interaction. To test this, we incubated equimolar amounts of recombinant purified His-Neph1-CD and His-ZO-1-PDZ1 proteins in the absence or presence of ISD (1 nM and 5 nM). Immunoprecipitation was performed using Neph1 antibody and the bound ZO1 was detected by western blotting using His antibody. A significant increase in Neph1-ZO1 interaction in the presence of ISD (1 nM) was observed as shown by densitometry analysis (Fig. 5A,B). In another assay, equimolar amounts of recombinant GST-Neph1-CD and His-ZO1-CD proteins were incubated in the absence or presence of ISD. As shown in the Fig. 5C, there was a gradual increase in Neph1-ZO1 interaction with increase in ISD concentration form 0.5 nM to 2.0 nM (Fig. 5C). This was further confirmed through an ELISA-based binding assay, where addition of ISD increased Neph1 and ZO-1 binding in a concentration dependent manner (Fig. 5D). In addition to the *in-vitro* binding assay, we tested whether ISD enhanced the interaction of endogenous Neph1 and ZO-1 proteins. Thus, ISD (1 µM and 5 µM) was added to the cultured human podocytes (the intracellular accumulation of ISD after 8 h was confirmed by mass spectrometry, where a measurable increase in ISD was noted in ISD treated podocyte cell lysate, when compared to the control cell lysate, supplementary Table 2) and immunoprecipitation was performed from the podocyte cell lysate using Neph1 antibody. Western blot analysis using ZO-1 antibody showed that similar to the *in-vitro* assay, presence of ISD (1 µM) significantly enhanced binding of endogenous Neph1 with ZO1 (Fig. 5E,F). It is to be noted that increasing ISD to 5 µM did not result in any further enhancement in Neph1 and ZO-1 interaction.

### ISD prevents injury-induced mislocalization of Neph1

**Figure 6:**
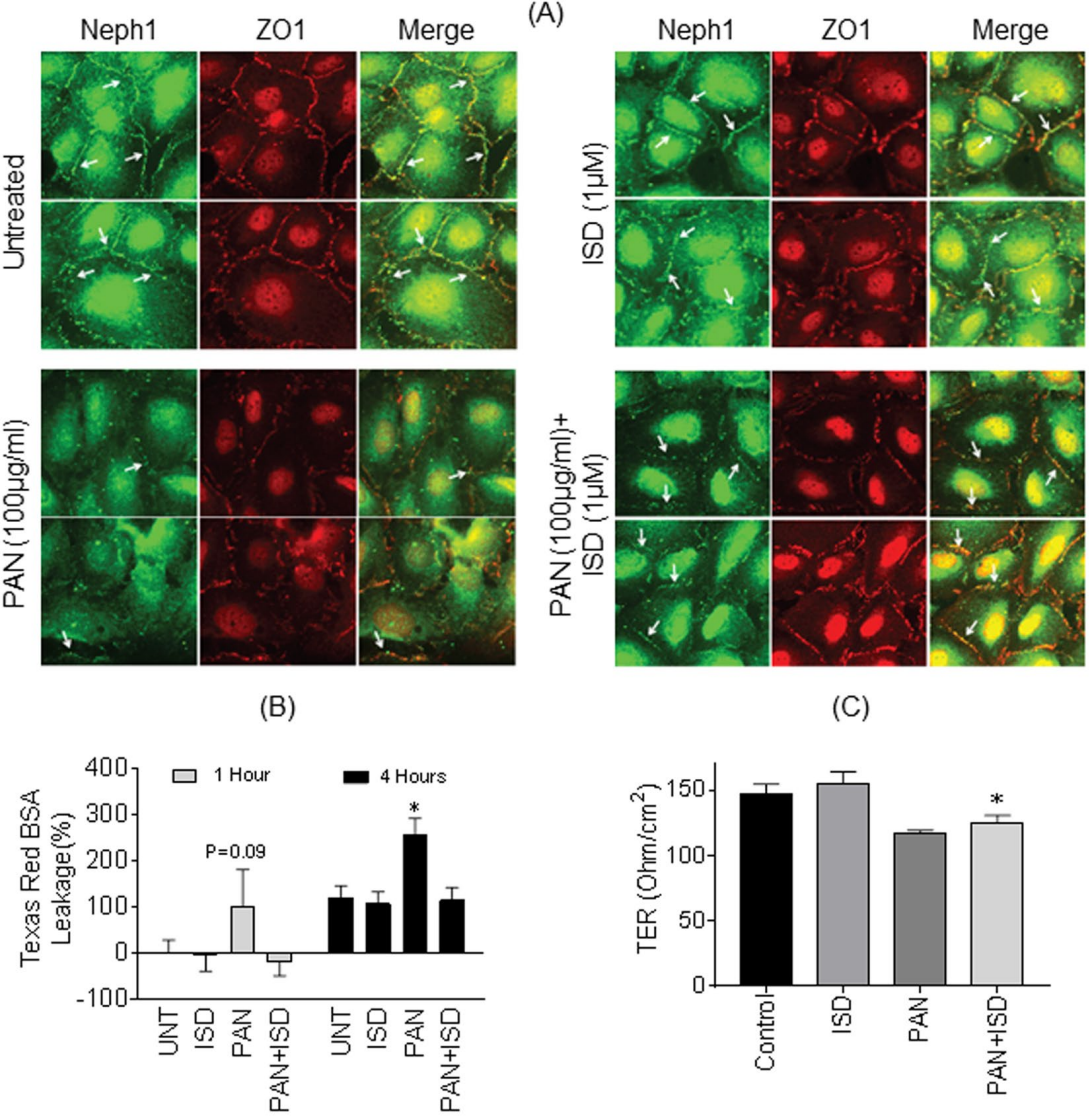
Treatment of cultured podocytes with ISD prevents injury-induced mislocalization of Neph1. (**A**) Cultured human podocytes were treated with PAN in the absence or presence of ISD and stained with Neph1 (Alexa-488) and ZO1 (Alexa-594) antibodies. Treatment with ISD prevented PAN-induced loss of Neph1 at cell-cell junctions (lower panel). (**B**) Effect of ISD on PAN-induced loss of transepithelial permeability was assessed by measuring the permeability of Texas Red-labeled BSA across the podocyte monolayer using trans-epithelial filter assay. The measurements were made after 1 h and 4 h of PAN injury and treatment with ISD significantly attenuated PAN-induced loss of permeability in these cells (P < 0.05). (**C**) Ability of ISD to restore PAN-induced loss of TER was measured in cultured human podocytes. TER was measured in a confluent monolayer of human podocytes treated with PAN (100 μg/ml) in the absence or presence of ISD (1 μM). ISD modestly restored PAN induced loss of TER in podocytes (p < 0.05).

Since ISD increased the interaction between Neph1 and ZO-1, we hypothesized that this may result in increased stabilization of Neph1 at the membrane. To test this hypothesis, we tested whether addition of ISD to cultured podocytes will prevent injury-induced mislocalization of Neph1^25,31^. Therefore, podocytes were grown on coverslips and subjected to treatment with puromycin aminonucleoside (PAN) (100 µg/ml) in the absence or presence of 1 µM ISD for a period of 48 hours. Immunostaining of cells with Neph1 and ZO1 antibodies showed that these proteins were localized at the cell-cell junction in untreated cells, whereas, the cells treated with PAN showed significant loss of Neph1 at the cell-cell junctions. In contrast, the cells that were treated with PAN and ISD retained the expression of Neph1 at cell-cell junctions (Fig. 6A), suggesting that ISD was able to prevent injury-induced mislocalization of Neph1 in these cells.

### ISD restored injury-induced loss of permeability and TER in podocytes

Since treatment with ISD preserved Neph1 localization at cell-cell junctions we wanted to further test if ISD can help maintain junctional integrity during injury. Therefore, we performed a transepithelial permeability assay where permeability of Texas Red-labeled albumin was measured across the podocyte monolayer. Podocytes were grown on transwell filters and treated with PAN in the absence or presence of ISD. The analysis of Albumin influx across the podocytes monolayer showed that PAN (100 μg/ml) induced an increase in albumin flux (which is an indicator of increase in transepithelial permeability) within 1 hour (p = 0.09), which significantly rose at 4 hour post treatment (p < 0.05); in contrast, addition of ISD (1 μM) prevented this increase in these cells at both time points (Fig. 6B). This was further confirmed by testing the ability of ISD to restore PAN-induced loss of electrical resistance in cultured human podocytes. Although ISD modestly restored the electric resistance, it was statistically significant (p < 0.05) (Fig. 6C). This suggests that ISD may help stabilize podocyte cell junctions such that they resist injury-induced damage.

### ISD attenuates Adriamycin induced death and glomerular phenotype in zebrafish

**Figure 7:**
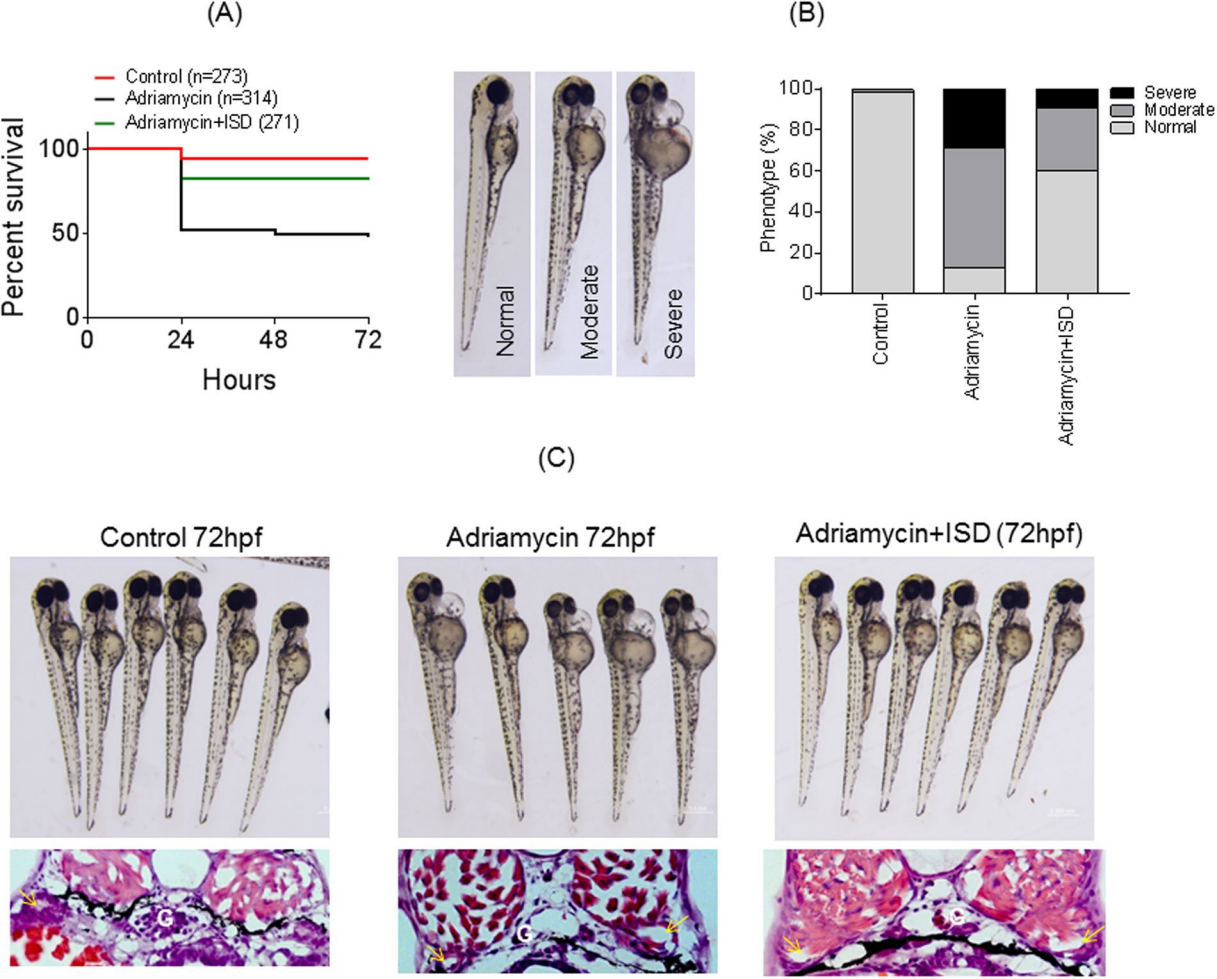
ISD attenuates Adriamycin induced death and glomerular phenotype in zebrafish: (**A**) Adriamycin (30.3 mg/liter) was added to zebrafish embryos (8hpf) to induce mortality and glomerular phenotype. The Kaplan-Meier survival curve shows a significant reduction in Adriamycin induced mortality in ISD treated fish (p < 0.05). (**B**) Adriamycin induced phenotype is categorized into normal, moderate and severe pericardial edema. Quantitative analysis suggests that ISD reduced moderate and severe phenotypes by more than 50%. (**C**) Histological analysis further revealed a significant reduction in the Adriamycin induced pronephric tubular dilation (a hallmark of renal injury, shown by arrow) and glomerular architecture damage (marked as G).

Adriamycin is known to induce pericardial edema extending to whole body, glomerular damage and death in zebrafish^32,33^. The Kaplan-Meier survival curve showed significant reduction in Adriamycin induced mortality in zebrafish upon ISD treatment. (p < 0.05) (Fig. 7A). In addition, the protection was noted in all phenotypes including severe and moderate pericardial edema (Fig. 7B). Quantitative analysis further suggests that ISD reduced moderate and severe phenotypes by more than 50% (Fig. 7B). Histological analysis further revealed a significant reduction in the Adriamycin induced pronephric tubular dilation (a hallmark of renal injury, shown by arrow) and glomerular architecture damage (marked as G in Fig. 7C).

### Treatment with ISD attenuates NTS-induced proteinuria in mice

**Figure 8:**
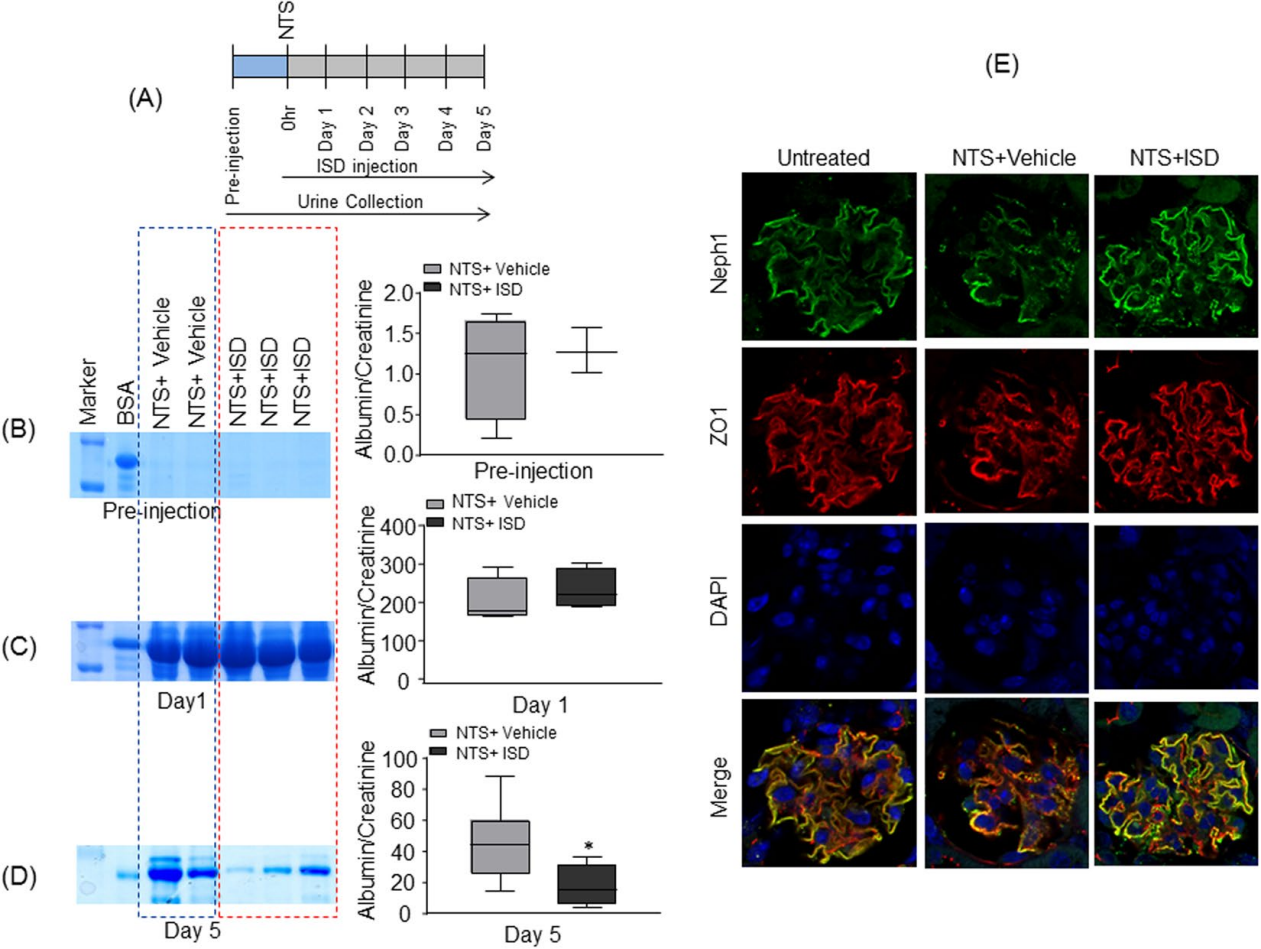
ISD attenuates NTS induced injury in mice: (**A**) The schematic presentation of experimental plan is shown. (**B**–**D**) Representative images of pre-injection, 24 hours and day 5 post NTS injection urine samples from NTS + vehicle and NTS + ISD mice as analyzed by SDS-PAGE and coomassie blue staining (left panel). Quantitative analysis (right panel) shows a significant decrease in urine albumin/creatinine ratio in mice treated with ISD at day 5 (p < 0.05), when compared to the vehicle control. (**E**) Immunofluorescence analysis of kidney sections by DAPI (Blue) and Neph1 (Green) and ZO1 (Red) antibodies shows NTS-induced mislocalization of Neph1 and ZO-1 (NTS + Vehicle), whereas treatment with ISD restored the colocalization of Neph1 and ZO-1 at podocyte cell membrane (NTS + ISD).

To further determine the *in vivo* applicability of ISD in preventing renal injury, we tested whether treatment of mice with ISD will induce protection from glomerular injury. Glomerular injury was induced in mice using NTS (nephrotoxic serum) as described previously^34^. ISD was injected into experimental mice at an interval of 24 h for the duration of experiment, whereas the control mice received equivalent amounts of saline. The mice were evaluated at pre-injection and at every 24 h for 5 days, by analyzing their urine samples using SDS-PAGE and albumin/creatinine ratios to estimate the degree of proteinuria. As shown in Fig. 8(A–D), while there was no difference in proteinuria levels at early time points, the mice that received ISD had significant decline in their proteinuria levels at 5 days when compared to controls. Original SDS-PAGE gels are included in the supplemental file. To further evaluate changes in the distribution of Neph1 and ZO-1, the kidney sections from these mice were analyzed by immunofluorescence using Neph1 and ZO-1 antibodies. While NTS induced mislocalization of Neph1 and ZO-1, treatment with ISD significantly restored their co-localization at the podocyte cell membrane (Fig. 8E). Collectively, these results provide compelling evidence that ISD mediated effect on Neph1 and ZO-1 interaction maybe therapeutically beneficial to podocytes; however, further investigations are needed to establish its therapeutic potential.

## Discussion

Since the incidences of kidney diseases are increasing worldwide, there is a greater need for novel therapeutic alternatives to prevent and preserve renal function during renal injury. Similar to other diseases, the pathophysiology of glomerular diseases also involves multi-protein complexes^28,30^. Targeting PPIs is a fast developing approach that has led to the identifications of many small molecules with potential to treat a variety of cancers^1,2,4^. While majority of these studies involve inhibiting the PPIs, we now show that promoting or strengthening a PPI may also have therapeutic advantages. In this proof of concept study, we targeted the Neph1 and ZO-1 complex that has been shown to participate in maintaining renal filtration function through its ability to stabilize podocyte junctions^10,11^.

There are many membrane proteins and their interactions that contribute to the structural integrity of podocyte junctions “slit diaphragm”^15,17,19,35^. Among these only a limited number of podocyte proteins including Neph1, Nephrin and ZO-1 have been structurally defined^11,26,36,37^. In addition to its interaction with ZO-1, Neph1 interaction with Nephrin may be critical for podocyte function^15,19^, as it has been shown to play a key role in maintaining podocyte integrity. While there are no known structures for Neph1-Nephrin complex, we previously published the structural details of interaction between Neph1 and ZO-1^11,26^. Our previous studies have shown that this complex is involved in podocytes response to injury, where injury induced dissociation of this complex and more importantly, these proteins reassociated during recovery from injury^10^. In addition to inducing the dissociation of Neph1 complex, glomerular injury also induces phosphorylation of Neph1 that initiates a downstream signaling cascade leading to actin cytoskeletal changes in podocytes^27,28^. When we targeted the cytoplasmic domain of Neph1, it inhibited the phosphorylation of Neph1, its displacement from the membrane and more importantly, prevented podocytes from injury^31^. This persuaded us to test the hypothesis that targeting Neph1 complexes that are mediated by its cytoplasmic domain may have similar therapeutic benefits. Since Neph1 and ZO-1 is a dynamic interaction, we hypothesized that a molecule that can simultaneously interact with these two proteins may affect the dissociation of this complex and stabilize this interaction.

While the interfaces of many PPIs can be large and flat, studies have shown that some PPIs interfaces may be compact enough and contain regions of “hot spots” where small molecules can bind^1,2^. To molecularly target a protein, it is imperative that its structural details have been characterized. Since we recently defined the structure of Neph1 and ZO-1 complex and provided insight into the molecular details of this interaction^11^, this allowed us to develop strategies to molecularly target this interaction. Structure-modeling analysis of this complex revealed the presence of a druggable pocket (where small molecules can bind), which was formed from contributions from both Neph1 and ZO-1 proteins. In silico screening of the pocket identified ISD as one of the potential candidates with ability to bind Neph1 and ZO-1 complex. It is interesting to note that ISD binding to Neph1 and ZO-1 complex primarily consisted of arginines, which is one of the most frequent amino acid in the hot spots of other PPIs^11^. The gel filtration analysis further suggested that addition of ISD (which received the highest screening score) to a mixture of Neph1 and ZO-1 proteins, created a stable complex that did not dissociate even after 48 h of incubation (Fig. 4). While other potential candidate molecules were also identified in this screen, none of them increased the stability of Neph1 and ZO-1 complex, suggesting that ISD may be the best fit molecule for this drug pocket.

ISD is a tetrafunctional pyridinium ring-containing amino acid that mediates the crosslinking of elastin polypeptide chains to form insoluble elastin fibers^38,39^. Its ability to significantly increase the binding between Neph1 and ZO-1 in the *in vitro* and *in vivo* experiments using purified proteins and podocyte cell lysate respectively, suggests that ISD may crosslink Neph1 and ZO-1 as predicted from structural modelling. Several studies have shown that ISD serves as a biomarker for elastin degradation in many inflammatory arterial diseases including chronic obstructive pulmonary disease (COPD), atherosclerosis, leukocytoclastic vasculitis and abdominal aortic aneurysm^40,41^. Thus it is freely available in human plasma and urine, and has been shown to be elevated in patients with lung damage and COPD^40,42^. Since our results demonstrate that addition of ISD protected zebrafish from adriamycin-induced injury and alleviated proteinuria in mouse model of glomerular injury, it raises the possibility that elevated amounts of ISD in lung patients may offer therapeutic benefits against glomerular diseases. However, this is a farfetched conclusion, since we only tested limited models of glomerular injuries, which may not represent a wide spectrum of glomerular diseases. Moreover, the amount of ISD used in our experiments was several folds higher than reported in patients where ISD biomarker studies were performed^40,42^. Thus more validation studies need to be performed before therapeutic claims for ISD can be made. Importantly, this study is primarily focused on presenting the nephrology community with a concept to identify novel molecules with therapeutic potential using structures of slit diaphragm proteins.

Our initial speculation was that molecules with the ability to stabilize Neph1 and ZO-1 interaction will be therapeutically beneficial in preventing podocyte injury. This was based on the premise that modulating Neph1 signaling and its injury-induced redistribution from podocyte cell membrane by the transduction of Neph1 cytoplasmic domain prevent podocytes from injury^31^. Since injury induces dissociation of Neph1 and ZO-1 complex and downstream Neph1 signaling^10,27,31^, binding of ISD to Neph1-ZO-1 complex may inhibit these injury-induced events thus affecting podocytes response to injury. Indeed, addition of ISD to podocytes prevented injury-induced loss of Neph1 from the podocyte cell membrane (Fig. 6), which may provide further support for this concept. However, detailed analysis of Neph1 signaling and trafficking events in the presence of ISD need to be performed to further highlight the mechanism for this protection. Although the results presented in this study suggest that ISD stabilizes Neph1-ZO-1 interaction, it remains to be understood how this translates into protection of podocytes from injury as seen in our cell culture experiment. Although the *in vivo* and mass spectrometry analysis (Supplemental Table 2) suggested that ISD is taken up by podocytes, the exact mode of ISD entry into the cells (whether it uses sodium dependent or independent transporters for cellular entry) also needs to be determined, which will further aid in developing second and third generation derivatives of this molecule with higher specificity and efficacy. In addition, such studies may highlight the mechanisms that participate in ISD mediated protection of podocytes and will form the basis of our future investigations. Apart from its interaction with ZO-1 there are other interactions of Neph1 that may be critical for podocyte function, such as its binding with Nephrin^25,28^. In addition, there may be several protein-protein interactions in podocytes that may be potential targets, but without the structural knowledge of these interactions, one cannot devise a strategy to molecularly target them. However, results presented in this study set a precedent for future studies where additional interactions of podocyte proteins can be structurally defined and molecularly targeted.

In addition to being easily available, compounds like ISD have minimal, if any side effects and thus higher chance of clinical success. While it is not our intention in this study to present ISD as a frontline therapy for treating any glomerular diseases, it does raise the possibility that there are natural molecules that may be therapeutically beneficial in treating glomerular diseases. With the availability of several commercial molecular libraries, we are in a position to screen additional molecules that would target Neph1-ZO-1 complex. Importantly, we show that in addition to cancers, renal diseases can also be targeted using this approach.

## Methods

### Docking studies

All the docking experiments were performed using the ICM software package^43^. The structural model of the complex of Neph1CD/ZO-1-PDZ1 described previously was used in this study^11^. The druggable pockets in the complex were identified using ICM’s Pocket Finder module^44^. Of the identified pockets, the pocket which lied at the interface of Neph1CD and ZO-1-PDZ1 was selected to dock a library of natural, un-natural and modified amino acids available with Sigma-Aldrich. The docking simulations were performed using Biased Probability Monte Carlo (BPMC) method. The grid maps were generated for the atoms in the chosen pocket which include hydrogen-binding interaction, electrostatic potential, van der Waals interaction and hydrophobic interactions. The docking runs were performed with fully flexible ligands which allowed calculation of energy of different poses of ligands within the identified pocket. Post-docking runs, the ligands were sorted by their calculated ICM-score which was summation of: 1) electrostatic energy, 2) hydrophobic energy, 3) hydrogen bond interactions, 4) internal energy of the ligand, 5) reduction of the entropy of ligand upon binding and 6) hydrogen bond donor and acceptor desolvation. The docking poses were visualized and the figures were generated for both the druggable pockets and lowest energy poses of molecules using the ICM program. The interactions made by ligands to the residues in the pocket were analyzed using programs Ligplot + ^45^ and server PLIP^46^. The 2D representations of the interactions were made using the program Ligplot + .

### Amino acids for interaction/stabilization studies

All the amino acids for the experimental purposes were purchased from Sigma.

### Neph1-CD and ZO-1-PDZ1 for gel filtration experiments

Both proteins, Neph1-CD and ZO-1-PDZ1 were expressed and purified as described before^11^. The protein mixtures were preincubated at 4 °C in the absence or presence of 5 μM ISD for various time intervals and injected into a gel filtration column. To test the effect of multiple ligands on Neph1-CD and ZO-1-PDZ1 interaction, the purified proteins were mixed in a molar ratio of 1:1 in the presence or absence of ligands to be tested. The mixtures were then injected into two Superdex 200 10/300 GL size exclusion columns attached in a sequential manner. Two columns were used in order to increase the resolution and clearly separate the peaks of Neph1CD/PDZ1 ZO-1 complex (~45 kDa) from Neph1CD (~35 kDa). The molecular weights of these too entities were too close to be separated using one size exclusion column.

### Antibodies and reagents

Neph1 antibody used in this study has been described previously^36^. Primary antibodies to ZO-1 (Invitrogen, Cat. No. 33–9100), His (Santa Cruz Biotechnology, Cat. No. sc-8036), and GAPDH (Sigma, Cat. No. G8795) were purchased commercially. The fluorophore secondary antibodies Alexa-Fluor-488 goat anti-rabbit (Invitrogen, Cat. No. A-11008), Alexa-Fluor-594 goat anti-mouse (Invitrogen, Cat. No. A-11005) were purchased from Invitrogen. All chemical reagents were commercially obtained from Sigma and/or MP biomedical.

### Pull-down experiments

The recombinant proteins His-Neph1-CD, His-Neph1-ΔPDZ, GST-Neph1-CD, and His-ZO1-PDZ1 were expressed and purified from Escherichia coli BL21 cells (Stratagene) as described previously^11^. Purified His-Neph1-CD or GST-Neph1-CD and His-ZO1-PDZ1 proteins (10 nM final concentration for each protein) were mixed in the presence or absence of Isodesmosine (ISD) (0.5–5 nM) and immunoprecipitation was performed using Neph1 antibody bound to protein G agarose or GST-beads. Binding between Neph1 and ZO1 was evaluated by western blotting using His-antibody. *In-vivo* binding between endogenous Neph1 and ZO1 proteins was performed in cultured human podocytes as described previously^10^. Podocytes were cultured in the presence or absence of two different concentrations (1 µM or 5 µM) of ISD for more than 24 hours. Cell lysate were prepared using RIPA buffer containing protease and phosphatase inhibitors and subjected to immunoprecipitation using Neph1 antibody. Immunoprecipitation complexes were analyzed by western blotting using ZO1 antibody. The developed blots were scanned in Adobe Photoshop. The cropped images from phototshop, showing relevant control and test protein bands were transferred to Adobe Illustrator, labeled. Brightness and contrast was adjusted throughout the images including the test and controls. No other manipulations were performed to alter the data in any form. The graphs were generated from excel data using graph prism software. Original blots are included in the supplemental file. The intracellular accumulation of ISD was analyzed through mass spectrometry. Briefly, human podocytes were incubated with ISD for 8 h in serum free medium. Following incubation, the cells were washed (3 times with 1XPBS and then 3 times with sterile water), and lysed by sonication. Lysate was submitted to Division of Nephrology, proteomics core facility for mass spectrometric analysis.

### ELISA

To analyze the effect of ISD on binding between Neph1 and ZO1, ELISA was performed using a previously published protocol with slight modifications^47^. ZO-1-PDZ1 was coated at a concentration of 150 ng in Sodium carbonate bicarbonate buffer (pH 9.5) in individual wells of a 96- well Maxisorp Immunoplate (Nunc, Rochester, NY) by incubating the plate at 4 °C overnight. The wells were blocked with 5% skimmed milk in PBS for 2 hours at room temperature. The plates were then washed thrice with 1X PBS/0.1% Tween-20 solution and once with 1X PBS. Neph1-CD was added to each well at a concentration of 150 ng per well with increasing concentration of ISD (0.1, 0.2, 0.5, 1 and 2 μm). In the control all proteins were added but no ISD and incubated overnight at 4 °C. The wells were washed 3 times with 1X PBS/0.1% Tween-20 solution and incubated with Anti rabbit anti Neph1 antibody at a dilution of 1:500 in PBS containing 3% BSA. Secondary antibody (HRP conjugated, Pierce) was added at a dilution of 1:10,000 in PBS containing 3% BSA, and the plates were kept for one hour at room temperature. The plates were then washed thrice with PBS-T, twice with PBS and developed by adding 100 µl of substrate (TMB) solution (Thermo Scientific). Incubation was done at room temperature and the reaction was stopped by adding 100 µl of 1 N H_2_SO_4_ and the absorbance was measured at 450 nm in a micro-plate reader (Molecular Devices).

### Cell culture and indirect immunofluorescence microscopy

The human podocyte cell line was cultured in RPMI medium supplemented with 10% fetal bovine serum, insulin-transferrin-selenium (ITS) supplement and 200 units/ml penicillin and streptomycin as described previously at 33 °C and 5% CO2^31^. Podocytes were treated with puromycin aminonucleoside (PAN) (100 μg/ml) in the absence or presence of 1 µM ISD for a period of 48 hours. The cells were washed with PBS and fixed with 4% paraformaldehyde (in 1 × PBS), followed by permeabilization with 0.1% SDS. Immunostaining was performed using Neph1 (Alexa-488) and ZO1 Alexa-594) specific antibodies. Fluorescence microscopy was performed using a Zeiss wide-field microscope keeping all parameters constant, including exposure time while collecting images. The images were processed using Image J software and transferred to Adobe Illustrator for labeling. Brightness and contrast adjustments were kept constant throughout the images.

### Albumin flux assay

The movement of Texas-red-albumin across the monolayer of cultured human podocytes grown on Transwell plates was investigated. The cells were treated with puromycin aminonucleoside (PAN) (100 μg/ml) in the presence or absence of ISD (1 μM) for a period of 48 hours. Texas red BSA at a final concentration of 50 μg/ml was added to the lower chamber of Transwell and the movement of Texas red labeled albumin to the upper chamber was monitored at 1 hour and 4 hours intervals. Fluorescence of Texas red labeled albumin in the upper chamber was measured at 590 nm excitation and 625 nm emission wavelengths. A standard curve was prepared from the serial dilutions of Texas Red-labeled albumin to calculate the amount of BSA moved from the lower to the upper chamber.

### Transepithelial electric resistance measurement

Human podocytes were grown in 12-well cell culture Transwell filters (0.4-μm pore; Corning) until they achieved complete confluence. Confluent monolayer of human podocytes was treated with puromycin aminonucleoside (PAN) (100 μg/ml) in the absence or presence of ISD (1 μM) for 48–72 hours. Transepithelial electric resistance (TER) was measured using an epithelial volt-ohmmeter (model EVOM; World Precision Instruments). The measured resistance was presented in ohms divided by the surface area (1.12 cm^2^) of the Transwell filter.

### Zebrafish model of renal injury

Wild-type zebrafish embryos were maintained and bred for experimental purposes at Zebrafish core faculty of the Medical University of South Carolina. The egg medium (E3) was used to grow zebrafish at 28.5 °C^48^. Adriamycin at a final concentration of 30.3 mg/liter was added to the E3 growth medium at 8hpf and incubated further for 48hpf to induce glomerular injury as described earlier (Kari G, 2007; Zennaro C, 2014). ISD (2 μM) was added along with Adriamycin at 8hpf and the phenotypic and histological evaluation was performed at 72hpf. The Kaplan-Meier survival curve was prepared using graphpad prism version 6 software.

### Nephrotoxic Serum mice model

All experiments were performed in 10–12 weeks old mice. Nephrotoxic serum (NTS) has been used to study acute glomerular injury in mice and protection from the ISD. Nephrotoxic serum was obtained commercially from the Probetex (PTX-001S). 100 μl of NTS was injected retro-orbitally as described previously^34,49^. Urine samples from individual mice were collected at pre-injection and every 24 hours post NTS injection for five days. ISD (5 μM) injection was simultaneously given to the experimental mice through the intraperitonial injection at every 24 hours for five days. Any urine sample that was contaminated with mice faeces was discarded. Urine samples were spun down at 4000xg for 5 minutes and then frozen at −80 °C for future experiments. All urine samples were run on 10% SDS-PAGE and examined by Coomassie blue staining. Urine albumin/creatinine ratios were obtained using an enzyme-linked immunosorbent assay (ELISA) Albuwell kit (Exocell) and creatinine companion kit (Exocell), and results were analyzed by an unpaired one-tailed *t* test (GraphPad Prism 7). The coomassie stained blots were scanned in Adobe Photoshop and cropped to show the relevant albumin bands for both test and controls. The images were transferred to power point, labeled and saved as tiff files. Albumin Creatinine data graphs were generated from the excel data using graph prism software. Immunofluorescence analysis of kidney sections were performed using Neph1 (Green) and ZO1 (Red) specific antibodies as described previously^36^. The imaging was performed using Olympus FV10i laser scanning confocal microscope fitted with 60 × 1.35 NA water-immersion objective. All imaging parameters including the exposure time were kept constant, throughout the procedure. Image processing was performed using the Image J software and all adjustments including the brightness and contrast were maintained constant throughout all images, the images were transferred to power point and saved as tiff files. All animal work was approved by the animal care committee of the MUSC and was performed as per the animal care guidelines.

## Electronic supplementary material

Supplementary Info
