# Supplementary material for: Targeting Neph1 and ZO-1 protein-protein interaction in podocytes prevents podocyte injury and preserves glomerular filtration function
Source: Sci Rep. 2017 Sep 21;7:12047. doi: 10.1038/s41598-017-12134-8 (PMC5608913; doi:10.1038/s41598-017-12134-8)

Targeting Neph1 and ZO-1 protein-protein interaction in podocytes prevents podocyte injury and preserves glomerular filtration function

Amin Sagar[#‡], Ehtesham Arif[#§], Ashish Kumar Solanki[§], Pankaj Srivastava[§], Michael G Janech[§], Seok-Hyung Kim[§], Joshua H. Lipschutz[§], Sang-Ho Kwon[§], Ashish*[‡] and Deepak Nihalani*[§]

[‡]CSIR-Institute of Microbial Technology, Chandigarh INDIA; [§]Division of Nephrology, Medical University of South Carolina, Charleston SC USA

# Both authors contributed equally.

*Address correspondence to: Ashish or Deepak Nihalani, CSIR-Institute of Microbial Technology, Sec 39A Chandigarh 160036 INDIA or College of Medicine, Medical University of South Carolina, Charleston SC 29425 USA Phone: +172-6665472 or 843-876-2372 Email: ashgang@imtech.res.in or nihalani@musc.edu

Supplementary Table 1: A list of interactions made by isodesmosine with Neph1 and ZO1.

**Hydrogen Bonds**				
**Index**	**Residue**	**Protein**	**H-A Distance**	**D-A Distance**
1	GLY25	ZO-1	2.12	2.94
2	PHE26	ZO-1	2.96	3.53
3	GLY27	ZO-1	2.27	2.99
4	PHE28	ZO-1	1.73	2.50
5	GLY29	ZO-1	2.35	2.98
6	ILE30	ZO-1	2.99	3.55
7	THR145	Neph-1	1.80	2.52
8	GLN226	Neph-1	3.60	4.06
9	GLN229	Neph-1	1.84	2.73
10	GLN230	Neph-1	2.58	3.54
11	GLN230	Neph-1	3.18	3.88
12	GLN230	Neph-1	2.73	3.33
**Salt Bridges**				
**Index**	**Residue**	**Protein**	**Distance**	**Chemical Group**
1	ARG22	ZO-1	4.51	Carboxylate
2	ARG90	ZO-1	4.39	Carboxylate
3	ARG214	Neph-1	3.49	Carboxylate
4	ARG231	Neph-1	4.33	Carboxylate

Supplementary Table 2

	Quantify Compound Summary Report														
	Printed Fri Jul 07 11:51:16 2017														
	Compound 1: Isodesmosine														
	#	Name	Typ	Std. Conc	RT	Area	IS Area	Response	Primary Flags	fmol/ul	%Dev	S/N	LOD	LOQ	
9	9	ISD_BLANKset2_20170624_5ul_v1			1	17.3		17.349	MM	1.48		2.83	1.5	2.2	BLOD
14	14	ISD_8hour_set2_20170624_5ul_v1			1	92.8		92.769	MM	2.888		11.2	1.6	2.5	>LLOQ
		Extrernal Cal Curve R^2 = 0.98													

LOQ is lower limit of quantification, LOD is lower limit of detection, BLOD is below limit of detection, S/N is signal to noise

Figure 5 Original Blots

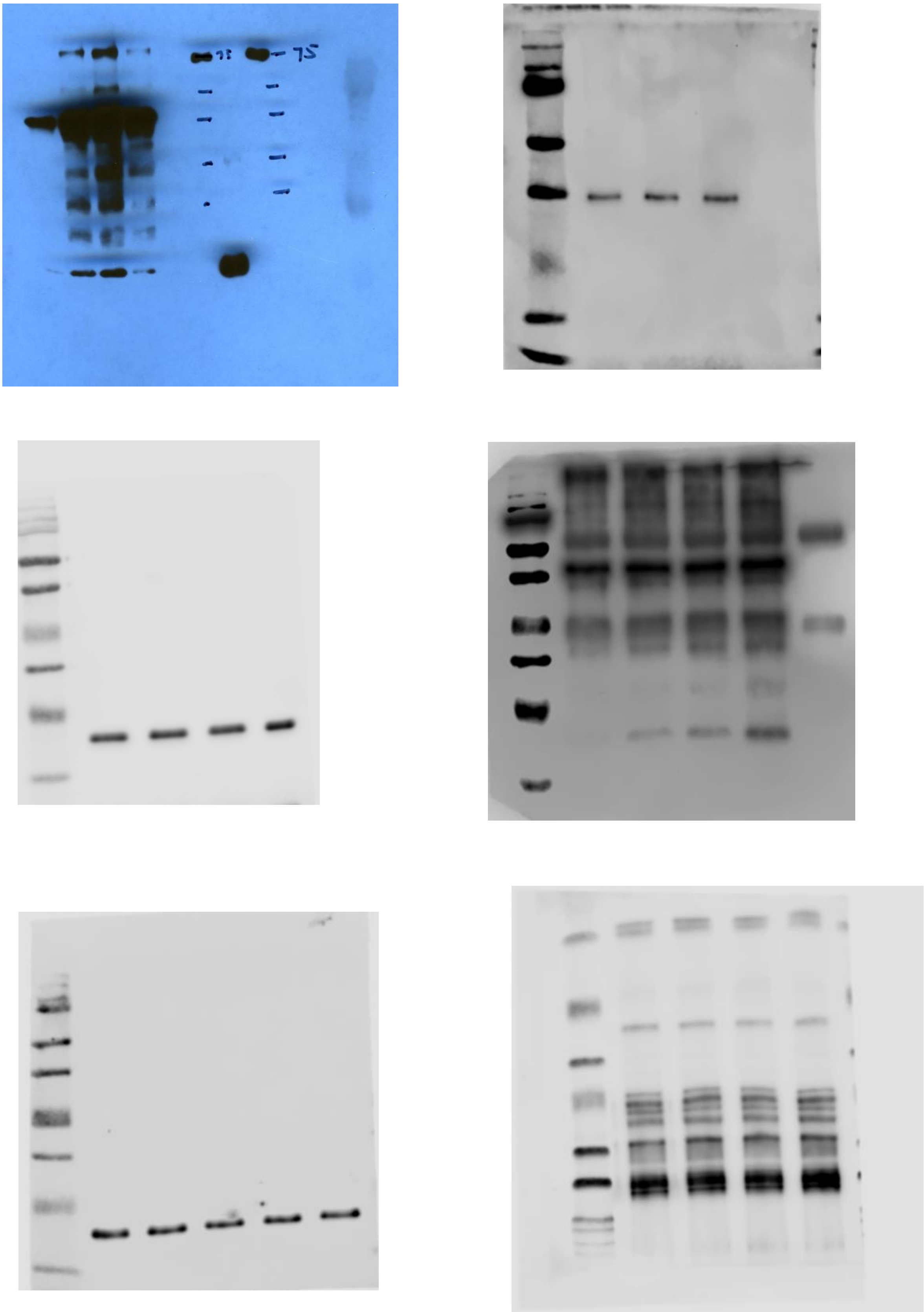

Figure 6 Original Blots

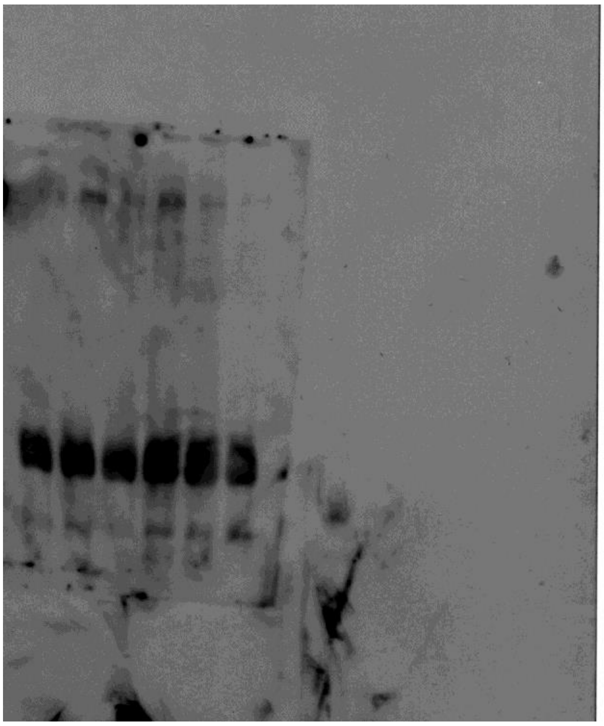

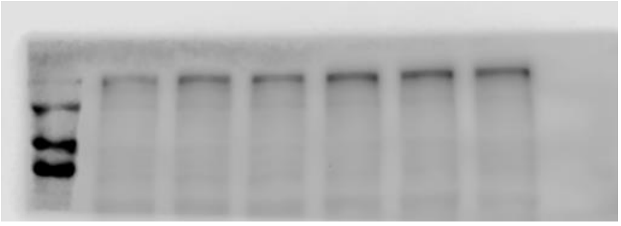

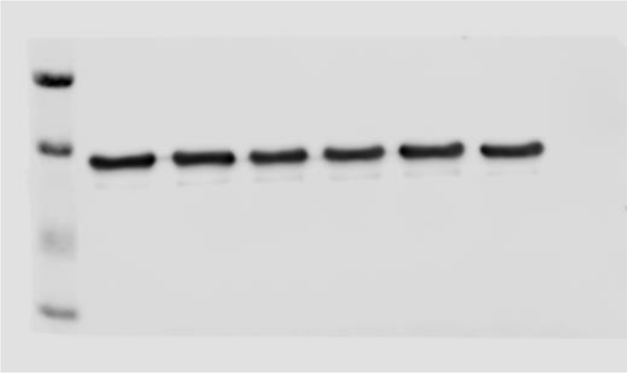

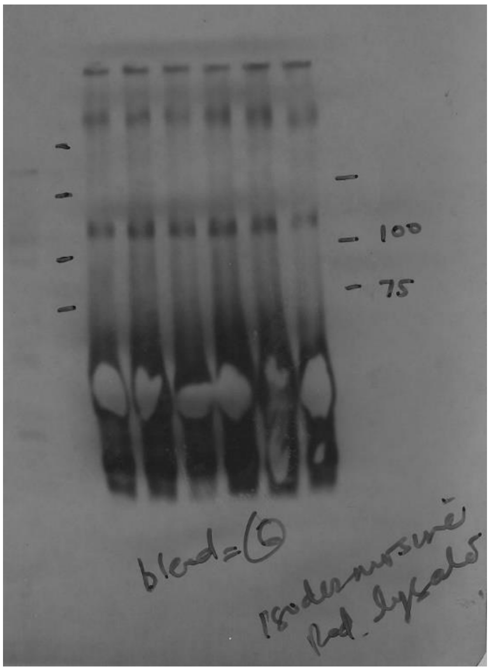

Figure 7 Original SDS –Gel pictures

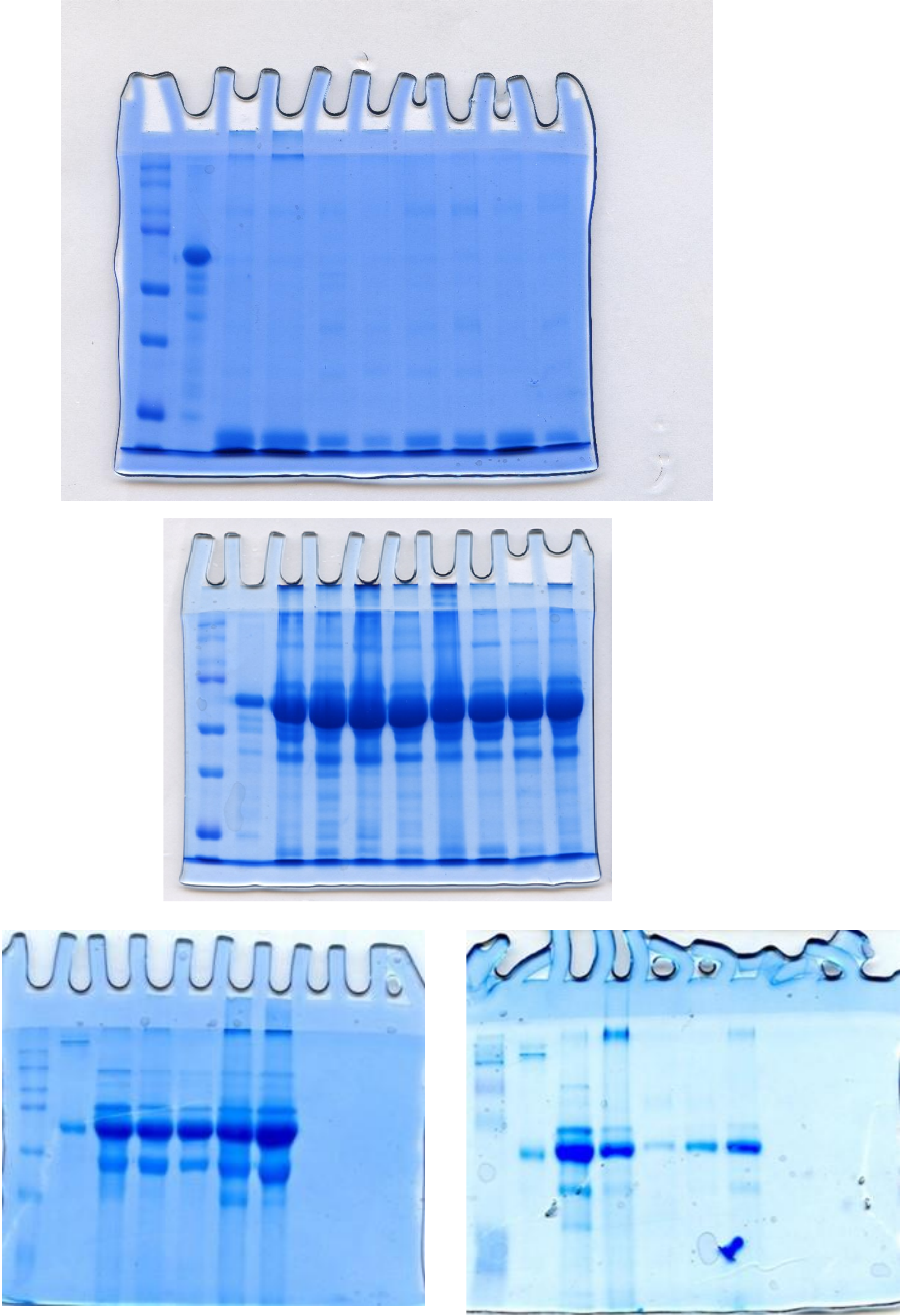

Supplement: Supplementary file 1 — Supplementary Info [file 41598_2017_12134_MOESM1_ESM.pdf]
